# Bacterial profiles of the oral, vaginal, and rectal mucosa and colostrum of periparturient sows

**DOI:** 10.1371/journal.pone.0317513

**Published:** 2025-02-12

**Authors:** Virpi Piirainen, Emilia König, Aleksi Husso, Mari Heinonen, Antti Iivanainen, Tiina Pessa-Morikawa, Mikael Niku

**Affiliations:** 1 Department of Production Animal Medicine, Faculty of Veterinary Medicine, University of Helsinki, Helsinki, Finland; 2 Department of Veterinary Biosciences, Faculty of Veterinary Medicine, University of Helsinki, Helsinki, Finland; 3 Research Centre for Animal Welfare, Department of Production Animal Medicine, University of Helsinki, Helsinki, Finland; University of South Carolina School of Medicine, UNITED STATES OF AMERICA

## Abstract

The commensal microbiota influences the health, feeding efficiency, and reproductive performance of sows. The microbiota composition in the alimentary and genitourinary tracts and in colostrum/milk during pregnancy and lactation also impacts the microbiota and immune system, growth, and health of the piglets. Knowledge of the microbial compositions is important for evaluation of these effects and for discovering ways to improve the health and productivity of the sows. Oral, vaginal, and rectal mucosa and colostrum were sampled from 32 sows of variable parity in late pregnancy, and colostrum within 6 hours of delivery of the first piglet, on four commercial piglet-producing farms in Finland. Microbial compositions were analyzed by 16S rRNA gene amplicon sequencing. The most abundant genera of the oral microbiota were *Rothia*, *Moraxella*, and *Streptococcus*. The rectal microbiota was dominated by *Clostridium sensu stricto 1*. *Streptococcus* was the most abundant genus in the vagina and colostrum. Moderate differences in diversity and composition were observed between farms. The relative abundances of the genera *Neisseria* (MaAsLin 2 q = 0.002, ANCOMBC q = 0.005), *Fusobacterium* (MaAsLin 2 q = 0.008, ANCOMBC q = 0.04) and *Bacteroides* (MaAsLin 2 q < 0.005, ANCOMBC q = 0.06) were lower in oral samples and *Romboutsia* (MaAsLin 2 q = 0.07, ANCOMBC q = 0.05), *Turicibacter* (MaAsLin 2 q = 0.08, ANCOMBC q = 0.02) and *Lachnospiraceae_UCG_007* (MaAsLin 2 q = 0.1, ANCOMBC q = 0.05) were higher in rectal samples of multiparous compared to primiparous sows. In vaginal samples there was a tendency of higher relative abundances of the genera *Fusobacterium* and *Streptococcus* in multiparous than primiparous sows. Among the differentially abundant taxa, *F*. *necrophorum* and *F*. *nucleatum* were identified in oral samples, *F*. *gastrosuis* and *F*. *necrophorum* in vaginal samples, and *S*. *dysgalactiae* in colostrum samples. This study provides a comprehensive overview of the mucosal and colostrum microbiota of periparturient sows during normal production conditions on Finnish commercial farms.

## Background

The mucosal surfaces, skin, feces, and glandular excretions of the mammalian body are all inhabited by distinct communities of resident microbes. Once established early in life, these microbiotas are relatively stable [1–3]. Commensal microbes provide health and wellbeing benefits to the host by improving maintenance of the epithelial barrier [4] and providing protection against pathogens [5]. They also influence host immune development and immune functions [6–9] and feed utilization [10]. Furthermore, commensal microbes have effects on neural development and function and may even impact host mood and behavior [11, 12].

Due to the potential benefits of the microbiota on the health and performance of the host, there has been a growing interest in the composition of the commensal microbiota of production animals, including pigs. These studies have mostly focused on growing pigs [13, 14], but the commensal microbiota of pregnant sows has also gained attention to an increasing extent [15–24]. Physiological factors, including phase of the reproduction cycle [16–19] and parity [21–23], can modulate the microbial compositions of adult animals. Environmental factors, such as diet, stress, and exposure to antibiotics [13, 25–27], can also have impacts. The fecal, vaginal, and colostrum (or milk) microbiotas of the sow in turn influence the developing microbiota and immune system of the offspring during pregnancy and lactation [6, 22, 24, 28, 29]. This has long-term effects on the health and growth of the piglets [6, 30]. Thus, knowledge on sow microbiota composition is valuable in evaluating its potential benefits and disadvantages to the offspring.

In this study, we explored the bacterial profiles of the oral, vaginal, and rectal mucosa and colostrum of 32 periparturient sows using 16S RNA gene amplicon sequencing. The study population included both primiparous and multiparous sows from four commercial Finnish piglet-producing farms. The bacterial profiles were compared between farms, parity groups, and individuals.

## Results

### Overview of sequencing data

16S rRNA gene amplicon sequencing of sow oral (n = 31), vaginal (n = 31), and rectal mucosa (n = 32) and colostrum (n = 31) samples resulted in 6 749 409 high-quality reads, with an average of 53 567 (± SD 12 960) reads per sample. The reads were mapped to 5379 amplicon sequence variants (ASV). Details of the sequencing data for each sample type are shown in S1 Table and results of the ZymoBiomics Microbial Community Standard positive control in S2 Table.

### Microbiota diversity in different mucosal sites

**Fig 1:**
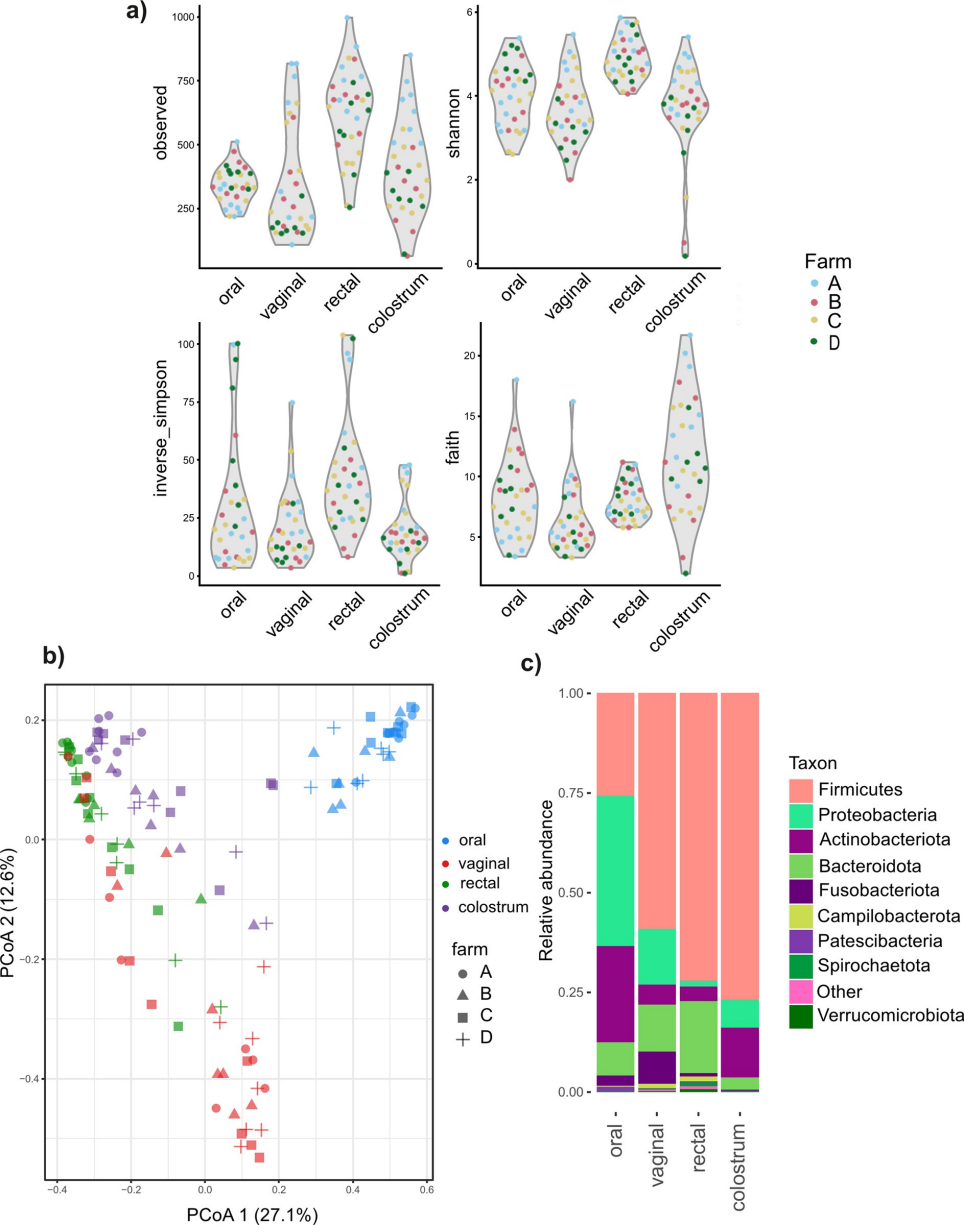
**Microbiota of 32 late-pregnant sows (oral, vaginal and rectal samples) and their colostrum during farrowing study sows originated from four Finnish commercial farms marked as A-D.** a) Alpha diversity: Observed richness, Shannon, Inverse Simpson and Faith indices, b) PCoA of Bray Curtis dissimilarities, c) relative abundances of major phyla.

The alpha diversity indices of the four sample types are shown in Fig 1A. Each sample type clustered separately in PCoA at the ASV level (Fig 1B; PERMANOVA p = 0.0001; for all pairwise comparisons, p.adj < 0.01). The oral samples were completely separated from the other sample types on the PCo1 axis. Vaginal samples were separated from the others on the PCo2 axis, with some overlap with the rectal cluster (Fig 1B). The colostrum samples clustered closest to but separate from the rectal samples and separate from the oral and vaginal samples (Fig 1B).

Nine bacterial phyla were identified in the oral samples, 11 in vaginal, 15 in rectal, and 11 in colostrum samples. Firmicutes, Bacteroidota, Proteobacteria, and Actinobacteriota were the dominant phyla in all sample types. In the oral samples, Proteobacteria were the most abundant phylum (38%), followed by Firmicutes (26%) and Actinobacteriota (24%). In the vaginal samples, Firmicutes (59%) were the most abundant phylum, followed by Proteobacteria (14%) and Bacteroidota (12%). In rectal samples, Firmicutes (72%) were the dominant phylum, followed by Bacteroidota (18%) and Actinobacteriota (3.7%). Firmicutes (77%) were also dominant in the colostrum samples, followed by Actinobacteriota (13%) and Proteobacteria (7.0%). The overall relative abundances (RAs) of the main phyla in each sample type are shown in Fig 1C.

At the level of prevalent bacterial species, oral and rectal samples had the largest numbers of species unique to these locations (S3 Table). Colostrum shared more species with rectal and vaginal samples than with oral samples.

### Sow oral microbiota

**Fig 2:**
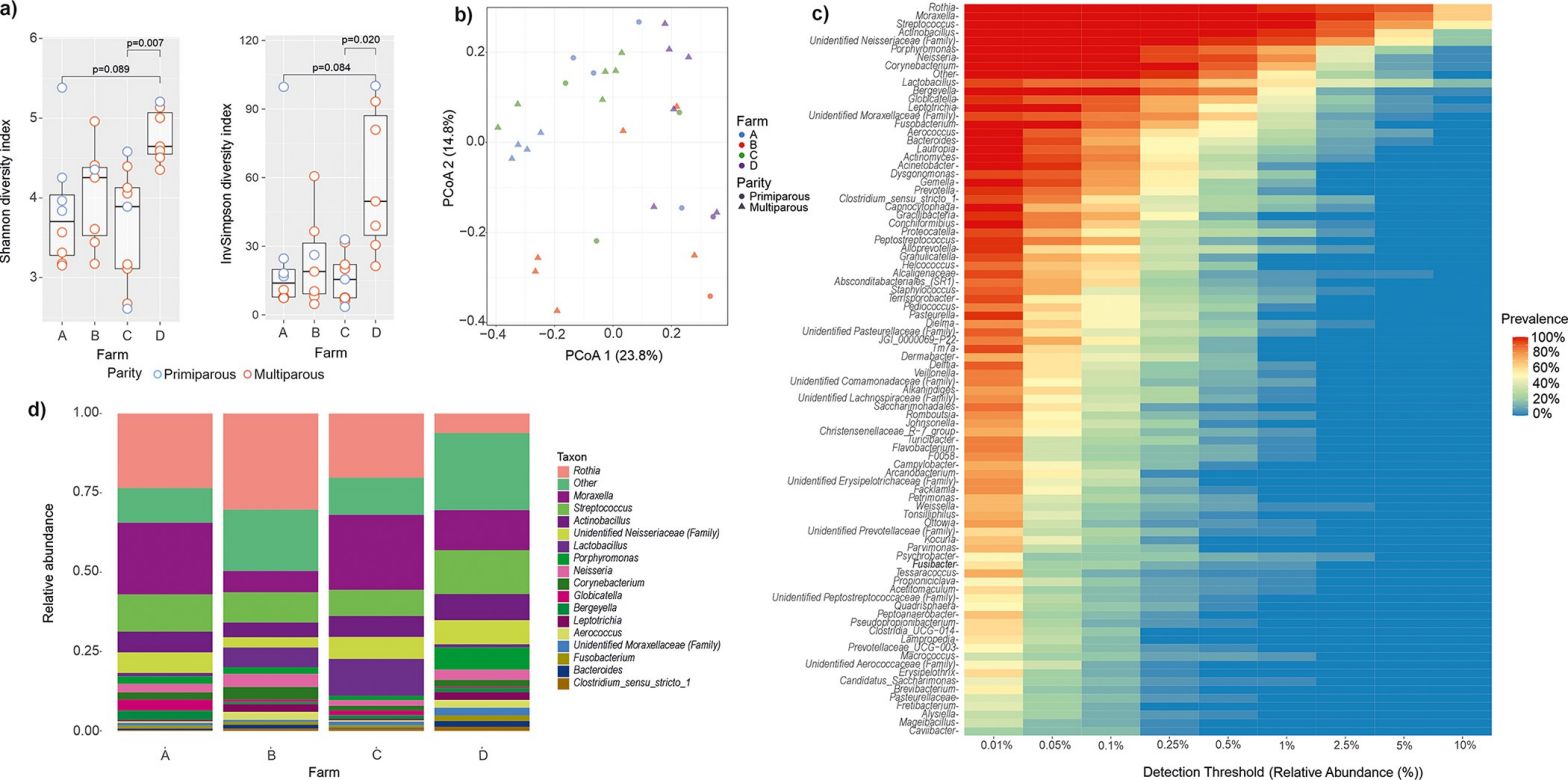
**Composition of the oral microbiota of 32 late-pregnant sows from four Finnish commercial farms marked as A-D.** a) Alpha diversity indices Shannon and Inverse Simpson. Bonferroni corrected p values are shown for pairwise comparisons using Wilcoxon rank sum exact tests, after a significant Kruskal-Wallis rank sum test. b) Principal coordinates analysis based on Bray-Curtis dissimilarities, c) Core heatmap, d) Average relative abundances of the main genera.

The median alpha diversity of farm D samples was higher than that of the others (Fig 2A and S1 Fig), but the difference was significant only between farms D and C in Shannon (p = 0.007) and inverse Simpson (p = 0.02). In PCoA, the samples from different farms overlapped, but a partial separation of farm D samples from the others on PCo1 and of farm B on PCo2 could be observed (Fig 2B).

In total, 113 genera were identified in the oral samples. The microbiota composition at the genus level is presented in Fig 2C. Twenty-one genera were present in all samples and constituted 79% of the total microbiota, calculated from the RA data. The most abundant of these genera were *Rothia*, *Moraxella*, *Streptococcus*, *Actinobacillus*, an unidentified genus of the Neisseriaceae family, *Porphyromonas*, *Neisseria* and *Corynebacterium*, all present with RA >0.1% (Fig 2C). There were some apparent differences in the genus-level compositions between the farms with farm A and D sows having higher RA of *Moraxella* and farm D lower RA of *Rothia* than sows from the other farms (Fig 2D). Further, sows from farms A and D had lower RAs of *Lactobacillus* than those of farms B and C (Fig 2D).

### Sow vaginal microbiota

**Fig 3:**
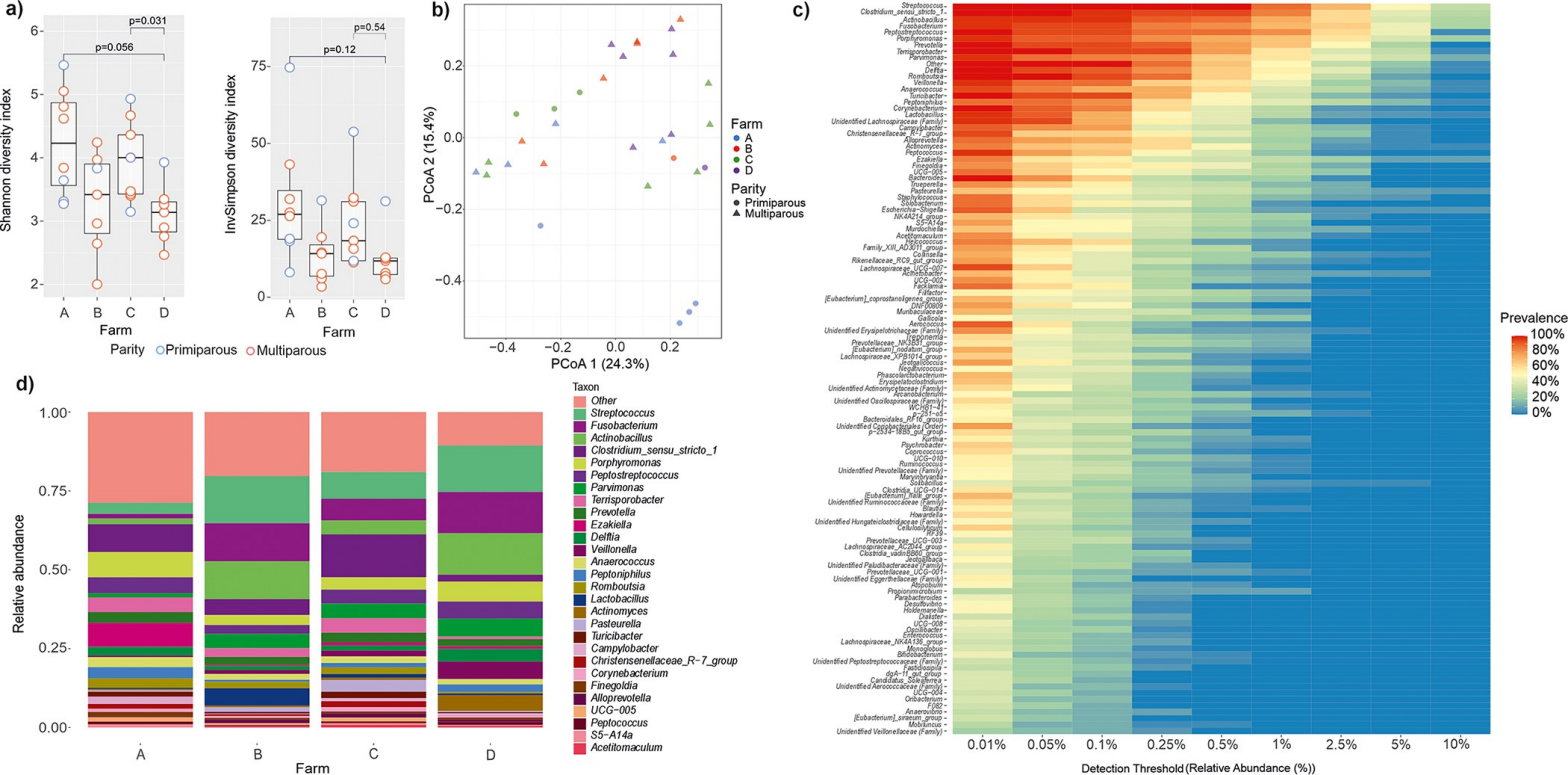
Composition of the vaginal microbiota of 32 late-pregnant sows from four Finnish commercial farms marked as A-D. a) Alpha diversity indices Shannon and Inverse Simpson. Bonferroni corrected p values are shown for pairwise comparisons using Wilcoxon rank sum exact tests, after a significant Kruskal-Wallis rank sum test. b) Principal coordinates analysis based on Bray-Curtis dissimilarities, c) Core heatmap, d) Average relative abundances of the main genera.

There were modest differences in the microbial diversities between the sows from the different farms, with farm A and C samples having higher alpha diversity than the others (Fig 3A and S1 Fig). Only the difference between farm C and D was significant in Shannon (p = 0.03). No differences were significant between the farms in Inverse Simpson’s. The farms were not separated in PCoA, but three primiparous sows from farm A clustered separately on PCoA 1 (Fig 3B).

In vaginal samples, 129 genera were identified. Altogether 13 of these were present in all samples constituting 42% of the total microbiota. *Streptococcus*, *Clostridium sensu stricto 1*, *Peptostreptococcus*, *Parvimonas*, *Terrisporobacter*, *Prevotella*, *Delftia*, *Romboutsia*, *Lactobacillus*, and *Turicibacter* were the most abundant of these genera (Fig 3C). *Actinobacillus* and *Fusobacterium* had also high RAs but remained undetected in one sample. Farm A samples had lower RAs of *Streptococcus*, *Fusobacterium*, and *Actinobacillus* than the others, but higher RAs of *Porphyromonas*, *Ezakiella* and *Peptoniphilus*. Farms B and D had higher RAs of *Streptococcus*, *Fusobacterium*, and *Actinobacillus* than farms A and C, and higher RA of *Lactobacillus* compared to other farms (Fig 3D).

### Sow rectal microbiota

**Fig 4:**
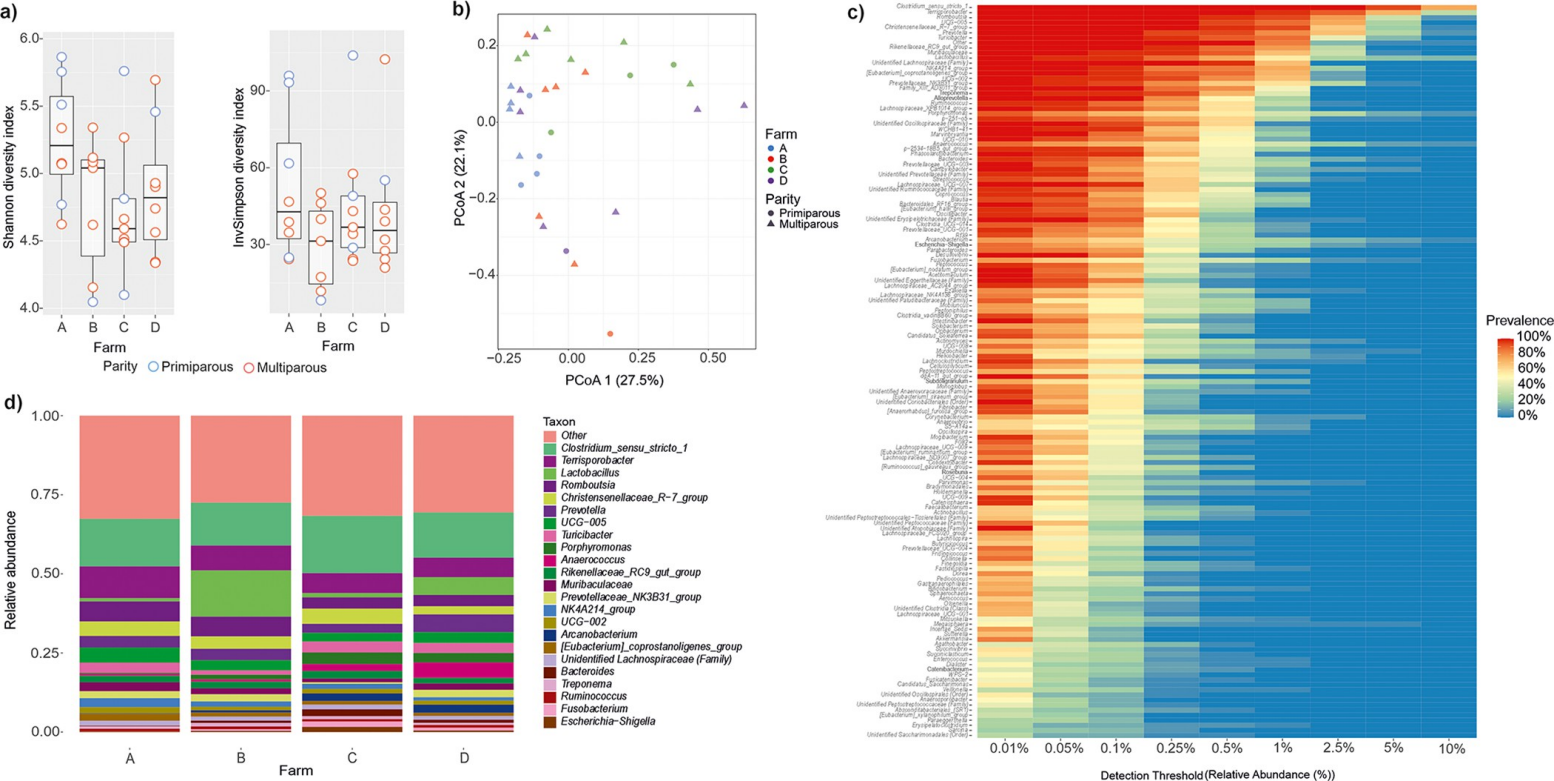
**Composition of the rectal microbiota of 32 late-pregnant sows from four Finnish commercial farms marked as A-D.** a) Alpha diversity indices Shannon and Inverse Simpson. There were no significant differences in the rectal sample alpha diversities between farms (Kruskal-Wallis rank sum test p = 0.15). b) Principal coordinates analysis based on Bray-Curtis dissimilarities, c) Core heatmap, d) Average relative abundances of the main genera.

Although the rectal microbiota in farm A was more diverse than that of the other farms (Fig 4A and S1 Fig), the differences in the diversity indices were not significant (p = 0.22 in Shannon, p = 1.00 in inverse Simpson). Farm C samples separated partially from farm A and farm B samples in PCoA, with overlap of all the three in the in top left corner, while farm D samples spread among all the other farm groups (Fig 4B).

Altogether 161 genera were identified in the sow rectal samples. Forty-one of these had 100% prevalence and accounted for 71% of the total abundance. The most abundant of these were *Clostridium sensu stricto 1*, *Terrisporobacter*, *Lactobacilllus*, *Romboutsia*, UCG-005, *Christensenellaceae*_R−7_group, *Prevotella*, *Turicibacter*, and *Rikenellaceae*_RC9_gut_group. Farm B samples had higher abundances of *Lactobacillus* and lower of *Turicibacter* than samples from the other farms (Fig 4D). The RA of *Lactobacillus* in samples from farm D was also higher than that of the samples from farms A and C. Farm A and B samples had more *Romboutsia* than the others (Fig 4D).

### Sow colostrum microbiota

**Fig 5:**
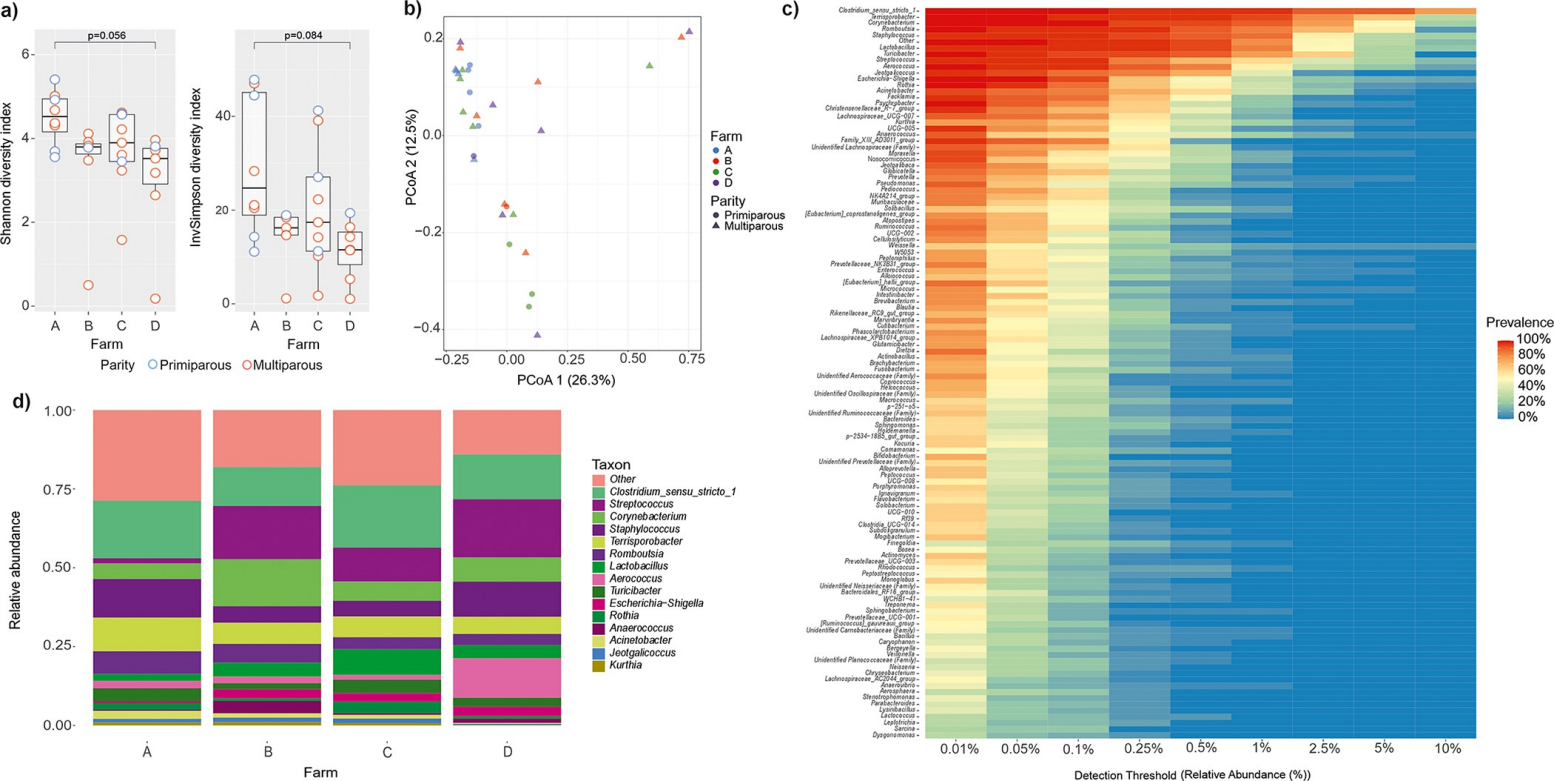
**Composition of the colostrum microbiota of 32 sows sampled during farrowing in four Finnish commercial farms marked as A-D.** a) Alpha diversity indices Shannon and Inverse Simpson. Bonferroni corrected p values are shown for pairwise comparisons using Wilcoxon rank sum exact tests, after a significant Kruskal-Wallis rank sum test. b) Principal coordinates analysis based on Bray-Curtis dissimilarities, c) Core heatmap, d) Average relative abundances of the main genera.

Although colostrum samples from farm A showed higher alpha diversity than others, the difference from the other farms was not significant (p = 0.056 for Shannon, p = 0.084 for Inverse Simpson) (Fig 5A and S1 Fig). In PCoA, most of the samples formed a cluster on PCo1, with three outliers belonging to two different farms clustering separately in the upper right corner (Fig 5B).

Among the 159 genera identified, 13 were present in all samples and accounted for 64% of the total abundance. *Clostridium sensu stricto 1*, *Terrisporobacter*, *Corynebacterium*, *Romboutsia*, and *Staphylococcus* were the most abundant genera (Fig 5C). Samples from farm A had lower abundance of *Streptococcus* and higher abundance of *Staphylococcus* and *Terrisporobacter* than other farms. Samples from farm B had higher RA of *Corynebacterium* and *Anaerococcus*, and lower RA of *Clostridium* than the others (Fig 5D). Farm C had more *Rothia* than other farms, and farm D more *Aerococcus* (Fig 5D).

### Parity effects on microbial compositions

**Fig 6:**
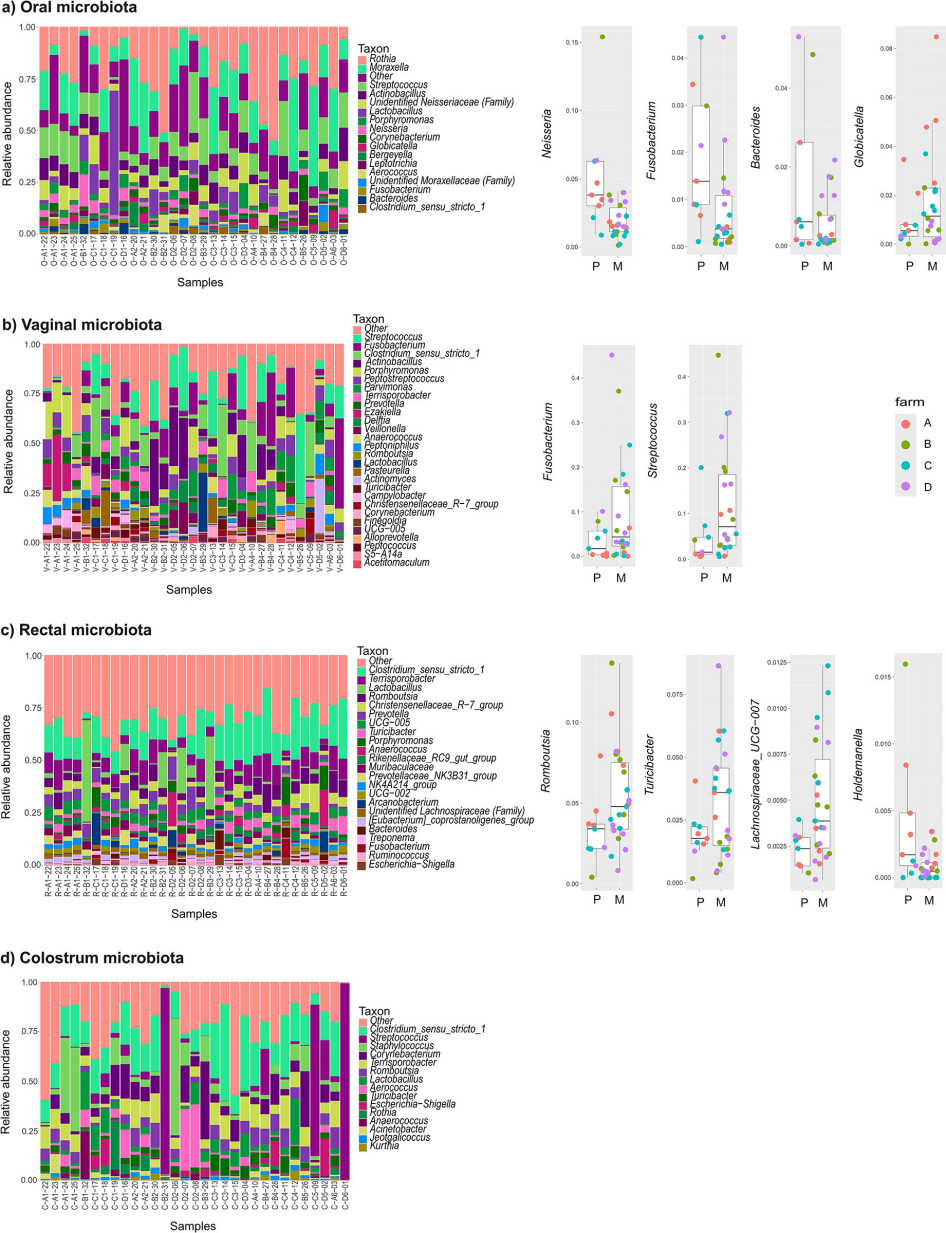
**Differences in the microbial compositions between primiparous (n = 9) and multiparous sows (n = 22) from four Finnish commercial farms marked as A-D.** a) oral, b) vaginal, c) rectal samples collected in late pregnancy, and d) colostrum during farrowing. Left: Relative abundances of major genera, samples sorted by parity (A1-D1 = primiparous, A2-D6 = multiparous). Right: Differences in relative abundances of specific genera between primiparous (P) and multiparous (M) sows. q values of the MaAsLin 2 and ANCOMBC tests are given in the text.

The alpha diversities in any of the four sample types were not significantly different between primiparous and multiparous sows, and the groups overlapped in PCoA (S2 Fig). However, we detected differences in the RAs of individual genera in each of the sample types. RAs of the main genera in all samples sorted by parity group are shown in Fig 6 (Left panel, a-d). Variation in the RAs of some of the major genera between individuals was extensive, especially of *Lactobacillus* in oral and rectal samples, *Fusobacterium* in vagina, and *Streptococcus* in colostrum. Distance-based redundancy analysis (dbRDA) indicated that parity significantly explained microbiota variation in mouth (p = 0.002) and rectum (p = 0.038) (S4 Table). Several genera in these sample types also showed differential RAs between primiparous and multiparous sows (Fig 6B Right panel a and c, S4 Table).

In oral samples, significantly lower RAs of the genera *Neisseria* (MaAsLin 2 q = 0.002, ANCOMBC q = 0.005), *Fusobacterium* (MaAsLin 2 q = 0.008, ANCOMBC q = 0.04) and *Bacteroides* (MaAsLin 2 q = 0.005, ANCOMBC q = 0.06) were observed in multiparous compared with primiparous sows whereas RAs of *Globicatella* (MaAsLin 2 q = 0.00008, ANCOMBC q = 0.05) were significantly higher in primiparous sows (Fig 6A Right panel). The lower RA of the genus *Fusobacterium* in the oral samples of multiparous sows was due to ASVs likely representing several species. Among these, *F*. *necrophorum* and *F*. *nucleatum* were unequivocally identified based on BLAST sequence comparisons; the others were unknown. ASVs likely representing several species of *Neisseria* had lower RAs in multiparous sows. These were unknown or the identification was ambiguous. The difference in *Bacteroides* was due to unknown species. No species were identified in the genus *Globicatella*.

In vaginal samples, there was a marked tendency towards higher RAs of the genera *Fusobacterium* and *Streptococcus* in multiparous compared with primiparous sows (Fig 6B Right panel). The difference in the RA of *Fusobacteria* was detected in the samples from all the four farms and for *Streptococcus* on three farms, although the differences were not statistically significant. 77% of the multiparous sows had higher RAs for Fusobacteria than the median value for the primiparous sows and 32% higher than any of the primiparous sows. The corresponding percentages for the sows with higher RAs of Streptococcus were 86 and 23%. The differences in *Fusobacteria* were due to ASVs that were identified as *Fusobacterium gastrosuis* and *Fusobacterium necrophorum*. The RAs of several species of the genus *Streptococcus* differed between the groups.

In rectal samples, significantly higher RAs of the genera *Romboutsia* (MaAsLin 2 q = 0.07, ANCOMBC q = 0.05), *Turicibacter* (MaAsLin 2 q = 0.08, ANCOMBC q = 0.02) *and Lachnospiraceae_UCG_007* (MaAsLin 2 q = 0.1, ANCOMBC q = 0.05) were observed in multiparous compared with primiparous sows (Fig 6C Right panel, S4 Table). The differences were observed in samples from all the four farms. *Holdemanella* was significantly more abundant in primiparous than multiparous sows (MaAsLin 2 q = 0.02, ANCOMBC q = 0.14), but the genus was absent in 80% of the sows in farm C. *Lachnospiraceae_UCG-009* (MaAsLin 2 q = 0.14, ANCOMBC q = 0.05), and *Clostridium_sensu_stricto_1* (MaAsLin 2 q = 0.14, ANCOMBC q = 0.009), were more abundant multiparous sows on all farms, but the differences were significant only in ANCOMBC (S4 Table). None of the ASVs with different levels in the two groups, corresponding to these genera, could be assigned to known species.

In colostrum, the RA of the genus *Streptococcus* was higher in multiparous than primiparous sows. This was due to colostrum samples from three multiparous sows having RAs of *Streptococcus* up to 75–98% (D6-01, B2-31 and C5-09 in Fig 6D Left panel). In addition, two samples (B4-27 and D5-02) had moderately high abundances of *Streptococcus* (20–22%). This was due to the high RA of an ASV matching the sequence of *Streptococcus dysgalactiae* in these samples. The average RA of this species in the other colostrum samples of multiparous sows was not higher than that of primiparous sows and remained < 0.2%. In the other sample types of the affected individuals the RA levels of *S*. *dysgalactiae* were not high, except for higher-than-average RA (16%) in the vaginal sample of sow D6-01. The vaginal RAs for the other affected individuals were all < 0,85% compared with < 1,5% of other individuals with *S*. *dysgalactiae* present in vagina. ASVs matching to *S*. *dysgalactiae* were not detected in any of the rectal samples.

## Discussion

The aim of this study was to obtain an overview of the mucosal and colostrum microbiota of sows on Finnish commercial farms under normal production conditions. The other studies in this field are from settings that were different with respect to geographical location, climate, animal breeds, and animal husbandry [15–21, 23, 24, 27, 29, 31–41]. We characterized the microbial communities present on oral, vaginal, and rectal mucosa of late pregnant sows and in colostrum from four piglet-producing farms. We detected differences between the farms both in microbial diversity and the RAs of genera. We also observed differences between primiparous and multiparous sows in the RAs of various genera in all the sample types. Some of these differences were shared between all the farms, while others were limited to only some of them.

Each of the four sampling sites, oral, vaginal and rectal mucosa, and colostrum, harbored distinct microbiotas that were significantly different from each other. The composition of the oral microbiota differed most from the others. Anatomical closeness may explain some of the similarities between rectal and vaginal microbiota. Colostrum microbiota has been suggested to be impacted by the intestinal microbiota via an endogenous entero-mammary pathway [23, 42] contributing especially anaerobic genera such as *Streptococcus*, *Lactobacillus* and *Bifidobacteria* to colostrum. There is however also evidence for an external route between intestinal and colostrum microbiota via environment and skin [42]. Our data does not allow us to make a distinction between possible endogenous or external interactions between the microbiotas.

Moderate differences between the farms were detected both in microbial diversity and RAs of the main genera in all the sample types. Many factors can contribute to differences in microbiota compositions, such as the genetic background of the sows [43, 44], details in feeding [25, 26, 45], antibiotic use and stress [27, 46]. Even though all the farms were equally representative commercial Finnish production farms using standard Finnish animal husbandry procedures, including feeding and raising of sows, it is not possible to rule out effects of some maintenance-related factors on the sow microbiotas. No differences were observed in the health of the sows between the farms. There was some variation in the sow genetic background between farms; however, while the sows on two farms (A and D) were of the same breed, the microbiotas of all the sample types in these two farms differed more in alpha diversity and composition compared to each other than compared to the other farms, suggesting that breed was not a central explaining factor for the observed differences. It is likely that other factors, such as characteristics of the physical and microbial environment [47], especially during early life when the microbiota of the sows was first established [24, 28, 48, 49] have contributed markedly to the formation of the microbial compositions of the sows on each farm.

### Oral microbiota

Proteobacteria was the most abundant phylum in the sow oral samples in contrast to the other sample types, which were dominated by Firmicutes. At the genus level, the oral microbiota was dominated by *Rothia*, *Moraxella*, *Streptococcus*, and *Actinobacillus*, followed by *Porphyromonas*, *Neisseria*, and *Corynebacterium*. Species belonging to these genera are commonly detected in the oral cavity and upper respiratory tract of various mammalian species, such as humans, dogs, and cattle [50–52]. Relatively few studies have addressed the composition of oral microbiota in pigs, mostly in piglets and growing pigs at various oral sites, such as tonsilla [53, 54], gingiva, buccal mucosa, and floor of the mouth [55]. The four top genera of our study were also identified as the most abundant in the saliva of sows and piglets in the study by Murase et al., where a similar procedure of sampling was used [32]. Overall, 10 of the top 20 genera were shared between the two studies. The composition in our study also overlapped with two other studies on sow oral microbiota where cotton swabs [24] or ropes [34] were used in sample collection, with eight of our 20 top genera shared with those of each of the studies. *Acinetobacter* was the dominant genus in both of these studies, *Streptococcus* was among the most abundant genera, but *Rothia*, *Moraxella*, and *Actinobacillus* were either less abundant or not detected. Only the genera *Streptococcus*, *Lactobacillus* and *Clostridium sensu stricto 1* were among the 20 most abundant in all the four studies, indicating variability of the RAs and prevalences of the oral genera. The differences in the reported compositions may be due to many factors, such as production and composition of saliva, and other host-related factors [56]. The sows in our study were not fasted before sampling, but oral samples were taken at a time when the animals we not offered feed. We cannot exclude the possibility that some of them may have eaten before sampling. However, composition of the oral microbiota does not appear to be directly dependent on timing of food intake, as saliva components and gingival crevicular fluid are the main nutrition sources for them rather than the feed, remaining only a short time in mouth [56]. Bacterial numbers in saliva have been shown to be unaffected by nutrient intake and remain the same in fed and fasted animals [57].

Detailed information on the effect of the reproductive cycle on the sow oral microbiota composition is not yet available. Li et al. reported a transient increase of *Actinobacillus* during parturition [24]. Pregnancy is known to induce changes in the human oral microbiota, some of which may be related to adverse pregnancy outcomes [58].

We detected significantly higher abundances of the genera *Fusobacterium*, *Neisseria*, *and Bacteroides* and lower of *Globicatella* in multiparous versus primiparous sows from the four farms. To the best of our knowledge, the effects of parity on the oral microbiota of sows have not been reported previously. The difference in *Fusobacterium* was attributed to *F*. *necrophorum*. *F*. *necrophorum* is present in the mouth, upper respiratory, and gastrointestinal tract and is an opportunistic pathogen in humans and other species [59, 60]. In pigs, *F*. *necrophorum* can cause necrotic stomatitis and facial necrosis [61, 62]. The species of *Neisseria* involved could not be determined without ambiguity. Several species of *Neisseria* are associated with periodontal health in humans [50] and dogs [51, 63]. *Bacteroides* is a genus of commensal bacteria in the alimentary tract utilizing plant derived sugars and producing metabolites that are useful for the host. The genus contains both pathogens and probiotic species [64]. *Globicatella* is a genus of sugar-metabolizing bacteria consisting of two species, *G*. *sanguinis* and *G*. *sulfidifaciens* occasionally identified in clinical infection samples in domestic animals [65].

### Vaginal microbiota

*Actinobacillus*, *Clostridium sensu stricto 1*, *Parvimonas*, and *Streptococcus* were among the most abundant genera in the vaginal microbiota. These genera have also been reported to belong to the most abundant in late-pregnant [27, 35] and mid-pregnant sows [36]. The compositions and RAs within the 10–20 most abundant genera vary between the studies. Notably, however, 19 (63%) of the 30 most abundant genera reported by Kiefer [35] in late pregnant sows were among the 30 most abundant in our study, and only 3 of them were not identified at all. Also, 20 of 32 (63%) most abundant reported from healthy sows of undetermined reproductive status [37] were identified within the 32 most abundant genera by us. *Actinobacillus* and *Clostridium* were also among the most abundant genera in weaning sows [66]. The RAs of *Lactobacilli* in our study were low compared to some studies [35, 37] but still within the range of RAs reported in other [27]. While *Lactobacilli* are dominant in the human vaginal microbiota and have an important role in the maintenance of vaginal pH, the RAs of *Lactobacilli* in other mammalian species, including pigs, are much lower [67, 68].

A marked proportion of the multiparous sows had higher RAs of the genera *Fusobacterium* and *Streptococcus* than primiparous sows. Although not statistically significant at the group level, this difference was seen in all farms and with 77% vs 86% of the multiparous sows having higher RAs of these genera than the median for the primiparous sows. The species corresponding to the differentially abundant ASVs were *F*. *gastrosuis* and *F*. *necrophorum*. *F*. *gastrosuis* is resident in the pig gastrointestinal tract and is implicated in gastric histopathology [69]. *F*. *necrophorum* is associated with metritis in dairy cows [70].

The vaginal microbiota is known to undergo changes during the estrous cycle, and pregnancy in human and cow [71, 72], less is known about microbiota changes during the reproductive cycle in sows. Bacterial taxa associated with normal estrus return have been identified in sows [66]. Parity-related differences in the sow vaginal microbiota have not been reported.

### Rectal microbiota

The most abundant genera observed in the rectal microbiota of sows from the current study include *Clostridium sensu stricto 1*, *Terrisporobacter*, *Lactobacilllus*, and *Romboutsia*. *Christensenellaceae*_R−7_group, *Prevotella*, *Turicibacter*, and *Rikenellaceae*_RC9_gut_group were also present in high relative abundances. These genera were also among the most abundant in other recent studies on sows from several geographically distinct locations and varying study designs [15–21, 23, 27, 33, 39]. There is variation across studies in the genera reported as the most abundant in sow fecal or rectal samples, with *Clostridium* as the most abundant in some studies [19, 23], including ours, and *Prevotella*, *Treponema*, or both in others [17, 21, 27], or one of the other major genera, such as *Lactobacillus* [20] or *Terrisporobacter* [39] in others. The genera defined as the minimal common fecal core in growing pigs by Holman et al. [73] (*Prevotella*, *Clostridium*, *Alloprevotella*, *Ruminococcus*, and the RC9 gut group) were among the most abundant both in our study and that of Shao et al. [33]. All the other studies reported at least 2 of them [15–21, 23, 27]. *Alloprevotella* was least frequently included. As for defining a minimal common core for the pregnant sows, comparing these 12 studies with 10–35 most abundant genera presented, there were no genera that were detected among the most abundant in all, but *Lactobacillus* (11/12), *Prevotella* (10/12), *Clostridium* (9/12), *Treponema* (9/12), and (*Rikenellaceae*_RC9_gut_group (8/12) were most commonly included. In addition to these, *Christensenellaceae*_R−7_group was among the most abundant in many of the sow studies [16, 19, 23, 27, 33, 39].

Parity was associated with the RAs of several genera in rectal samples. Differences included a significantly higher RA of *Turicibacter*, *Romboutsia*, and *Lachnospiraceae*_UCG_007 and lower RA of *Holdemanella* in multiparous compared with primiparous sows. The bacteria belonging to the genus *Turicibacter* are prevalent mammalian gut commensals with effects on host serum lipid and bile acid profiles [74]. In late pregnant sows, *Turicibacter* is one of the genera that shows increased RA by addition of indigestible fiber to the feed and its RA correlates with higher levels of host serum butyrate and lower of the proinflammatory cytokine IL-6 [39]. Bacterial species belonging to the genus *Romboutsia* are gastrointestinal or environmental anaerobes identified from animal and human ileal samples [75, 76]. Little is known of their role in the gastrointestinal tract, but higher RAs of *Romboutsia* along with other commensal intestinal genera are associated with lower prevalence of type 2 diabetes and lower RAs with hypertension in humans [77, 78]. *Lachnospiraceae* and *Holdemanella* are producers of short-chain fatty acids [79, 80].

Other genera with a tendency of higher RAs in multiparous shows detected on all farms included *Clostridium sensu stricto 1* and *Prevotella*. Higher RA of *Clostridium sensu stricto 1* in third parity sows compared with first parity sows has also been reported earlier [23]. *Clostridium* species are commensal bacteria known as butyrate producers in the gut [81]. *Prevotella* contains species with the ability to degrade plant glycans [82]. In growing pigs, it is a dominant genus that has multiple interactions with other microbial taxa; *Prevotella* species have effects on feeding efficiency [83]. Higher RAs of Prevotellaceae and Bacteroidota in high vs low parity [21] and of the genera *Prevotella* and *Enterococcus* in third parity [84] have been observed previously.

The differences in the abundance of the genus *Lactobacillus* detected in our study were due to only a few individuals and could not be considered as consistent over the data. Parity-related differences in the abundances of *Lactobacillus amylovorus* and *L*. *reuteri* have however been observed in pregnant sows by Berry et. al [22]. They also observed increased abundance of *Treponema bryantii* with higher parity. The genus *Treponema* was detected in our study, albeit with lower RA than in some other studies, and its RA did not differ between the parity groups. Several species of *Treponema* were present, but no ASVs corresponding to *T*. *bryantii* were detected.

### Colostrum microbiota

The most abundant genera in colostrum were *Clostridium sensu stricto 1*, *Streptococcus*, *Staphylococcus*, *Corynebacterium*, *Terrisporobacter*, *Romboutsia*, and *Lactobacillus*. *Clostridium* and *Lactobacillus* were identified among the most abundant genera also in all the other studies compared and *Corynebacterium* and *Streptococcus* in all but one [23, 29, 31, 38, 40]. *Terrisporobacter*, *Romboutsia* and *Staphylococcus* were also frequently identified as one of the major genera [28, 38]. The overall similarity of the reported ten most abundant genera between our study and the other studies was at least 20–48% [29, 31, 40] and 70% at the highest [23, 38]. *Pseudomonas* was reported as the core genus by Li et al. [24], with variation between the study breeds for the abundances of other genera. According to feeding experiments, changes in sow diet during late gestation and lactation may induce differences in colostrum microbial composition [40], but the differences did not involve the main genera discussed above.

The main reason for the variation in colostrum samples between the parity groups was the high RA of the genus *Streptococcus* in five (of 22) multiparous animals from three different farms. This was due to the exceptionally high RA of the species *S*. *dysgalactiae*. This species is a known pathogen that causes mastitis in cows [85] and has been found in increased abundance in sows with purulent vaginal discharge [86]. When transmitted to newborn piglets it may cause arthritis and encephalitis [87]. The source of *S*. *dysgalactiae* in the colostrum of the study sows remains unknown. It was present in vaginal samples of both affected and nonaffected individuals but was not detected in rectal samples. Environmental transmission is also possible [85].

### Limitations of the study

This was an exploratory study on the compositions of mucosal and colostrum microbiota on sows reared under regular commercial production conditions. It was not specifically designed to study differences between sows of different parity. Therefore, the results presented must be considered preliminary. However, it is notable that our analysis uncovered differences between the rectal microbiotas of primiparous and multiparous sows that are similar to those previously reported from study settings specifically designed for that purpose.

## Conclusions

Our study presents the microbial compositions of the mucosal sites and colostrum of commercially reared pregnant sows from Finnish piglet-producing farms. Diverse microbiotas with some variations between farms were discovered, with *Rothia*, *Moraxella*, and *Streptococcus* as the major genera in oral, *Streptococcus* in vaginal, *Clostridium sensu stricto 1* in rectal, and *Streptococcus* in colostrum samples. Several potentially interesting differences between primiparous and multiparous sows were detected, involving *Fusobacterium* and *Neisseria* in oral, *Fusobacterium and Streptococcus* in vaginal, *Turicibacter*, *Romboutsia*, *Lachnospiraceae_UCG_007* in rectal, and *Streptococcus* in colostrum samples. Further targeted studies are needed to define the differences related to parity and the impact of the sow microbiota on reproductive health.

## Materials and methods

### Ethics approval

The experiment was conducted under the EU legislation on the protection of animals used for scientific purposes (Directive 2010/63/EU) and approved by the Southern Finland Regional State Administrative Agency (ESAVI/16950/2018). The farm owners provided signed written informed consent at the beginning of the study.

### Farms, animals, and management

Four commercial piglet-producing farms in western and southwestern Finland participated voluntarily in the study during 2018–2019. The study farms are hereafter referred to as farms A-D. All study farms belonged to the health classification register for pig farms in Finland [88] which requires freedom from swine enzootic pneumoniae, atrophic rhinitis, swine dysentery, sarcoptic mange, and salmonellosis.

Detailed information about expected farrowing days and sow parities of one farrowing group in each farm were obtained from the farmers. The first farm visit was planned to enable the researchers to supervise the maximum number of farrowings in three days on each farm. Otherwise, the farm staff followed their normal management practices during the study. The researchers inspected the study sows visually while supervising their farrowings. None of the sows were considered to have health concerns.

On each farm, the sows in the farrowing units were housed in farrowing pens (4.6–4.8 m^2^) with crates and partly slatted floors. No bedding material was used for the sows and piglets in the farrowing pens. Within the farrowing pen, the piglets had a separate nest with a heat lamp and solid floor. The sows were fed a standard barley-based liquid feed three times a day in farms A-C and in farm D three to four times before farrowing, twice right after farrowing, and four times daily during the rest of the lactation. Detailed composition of the feed was not available to the researchers. Further details of the study farms, animals, and sow management are shown in Table 1.

**Table 1:** Descriptive information about the four study farms, animals, and management.

Farms and animals					Management			
Farm	Total number of sows in farm	Total number of study sows, primiparous (P) and multiparous (M)	Parity of study sows, median (range)	Breed of study sows	Sows moved to farrowing unit, days before farrowing	Nest-building material given prior to expected farrowing	Supervision (S) of farrowings and use of oxytocin (O) around farrowing	
A	940	8 P: 4 M: 4	2, (1–6)	Topigs Norsvin	5	Max. 1 L of straw occasionally, not routinely		S: no O: to all sows after the birth of the last piglet
B	450	7 P: 1 M: 6	3, (1–5)	Landrace x DanBred	3	Max. 1 L of hay and straw, 3 days prior, less frequently than daily		S: during working hours O: to all sows during farrowing
C	450	9 P: 3 M: 6	3, (1–5)	DanBred	5	1–5 L hay once a day, 5 days prior to farrowing		S: during working hours O: if problems with milk let-down or expulsion of placentas
D	1100	8 P: 1 M: 7	2, (1–6)	Topigs Norsvin	5	Max. 1 L of straw once a day, 5 days prior to farrowing		S: during working hours O: in case of delayed expulsion of placentas

### Sampling

Swab samples from sow oral, vaginal, and rectal mucosa were collected from 32 pregnant sows on average 1.5 (SD 1.2) days prior to farrowing. Factory clean protective gloves were used during sampling. Sterile flocked swabs (FLOQSwabs®, Copan Diagnostics Inc, CA, USA) were used for oral and vaginal samples to ensure sufficient material to be collected for the microbial analysis. Oral samples were taken by wiping the buccal mucosa, and vaginal samples by opening the vulva with one hand and wiping the mucosa just inside the vagina with the swab. From one sow, the oral sample was not obtained. Rectal samples were taken with sterile cotton swabs (Applimed SA, Châtel-St-Denis). The swabs were placed into cryotubes that were placed in a cool box, moved to a –18°C freezer within an hour and further to a –80°C freezer on arrival at the laboratory. Colostrum samples were milked within 6 hours from birth of the first piglet into sterile 10-ml plastic tubes after disinfection of the udder as described in detail in [89]. From one sow the colostrum sample was not obtained. Colostrum samples were stored similarly as the other samples.

### DNA extraction

DNA from all sample types was extracted using a ZymoBIOMICS^TM^ DNA Miniprep Kit (Zymo Research, Irvine, CA) with minor modifications to the kit instructions as described below. Different sample types were processed at different times to avoid cross-contamination between samples.

All samples were thawed on ice on extraction day. The sampling swabs were transferred with sterile instruments into lysis tubes (ZR BashingBead Lysis Tubes, Zymo Research, Irvine, CA), and 750 μl of ZymoBiomics Lysis Solution was added to each tube. Bacterial cells were lysed three times with a Fastprep® 24 (5.5 m/s, 60 s); the tubes were then centrifuged 16000 x g for 5 min at 4°C. The supernatant was transferred into ultraclean Eppendorf tubes and 200 μl of lysis solution was added to the tubes. The fast-prep and centrifugation steps were repeated. The supernatants were combined and centrifuged at 8000 x g for 1 min at room temperature. Plain sterile flocked swabs (FLOQSwabs®, Copan Diagnostics Inc, CA, USA) were used as negative controls for vaginal swabs during three of four analysis rounds. For oral and rectal swabs, negative controls were not included in the extraction round.

For colostrum samples, 2 ml of thawed sample were transferred into ultraclean microcentrifuge tubes and centrifuged at 16000 x g for 10 min at +4°C. Excess supernatant was removed until 200 μl of the supernatant remained. 750 μl of lysis solution (ZymoBiomics Lysis Solution) and 19 μl 20 mg/ml proteinase K solution in storage buffer (Zymo Research, Irvine, CA, USA) were added into each tube and mixed. The tubes were incubated at 55°C for 30 min. The remaining steps of the extraction from fast-prepping onward were performed as for the other sample types. Ultrapure water (Zymo Research, Irvine, CA, USA), stored in ultraclean microcentrifuge tubes in a similar way to the colostrum samples, was used as a negative control for colostrum samples.

After extraction, the DNA concentration of all sample types was measured with Qubit 3.0 Fluorometer (Thermo Fisher Scientific Inc., Waltham, MA).

### Library preparation and 16S rRNA gene amplicon sequencing

The hypervariable regions V3-V4 of the 16S ribosomal RNA (rRNA) genes were sequenced using the Illumina MiSeq platform in the DNA core facility of the University of Helsinki, essentially as described previously [90, 91]. Samples, negative controls, and ZymoBiomics Microbial Community Standard (Zymo Research, Irvine, CA, United States) were first amplified using 1× Phusion Hot Start II High-Fidelity PCR Master Mix (Thermo Scientific), 2.5% DMSO (Thermo Scientific), 500 nM of 16S rRNA V3 and V4 gene primers (341F and 785R; Metabion), and 1.25 μl of DNA extract. Total volume was 25 μl. The thermal cycling conditions included an initial denaturation step at 98°C for 30 s, 14–21 cycles of denaturing at 98°C for 10 s, annealing at 56°C for 30 s, and extension at 72°C for 20 s (14 cycles for fecal samples, 18 cycles for oral and vaginal samples, and 21 cycles for colostrum samples). The final extension step was at 72°C for 5 min. A T100™ Thermal Cycler (Bio-Rad Laboratories) was used. The amplicons were treated with exonuclease I and shrimp alkaline phosphatase for 30 min at 37°C to remove excess free primers. The second-round PCR amplifications were performed using an Illumina forward and reverse primer set, Phusion Hot-Start II polymerase, High Fidelity buffer, and 2.5% DMSO. The following thermal cycling conditions were applied with an Arktik thermal cycler (Finnzymes/Thermo Scientific): initial denaturation at 98°C for 30 s, 18 cycles at 98°C for 10 s, 65°C for 30 s, 72°C for 10s, and a final extension at 72°C for 5 min. Sample libraries were then pooled, and the pool was purified with a bead wash (MagSi-NGS Plus 0.9x). The final 16S rRNA gene amplicons were sequenced on an Illumina MiSeq sequencer using the v2 600 cycle kit paired-end (325 bp + 285 bp).

### Bioinformatics and statistics

The raw sequence data were processed as described in detail previously [91]. Briefly, the read quality was inspected using FastQC and MultiQC [92, 93]. Primers and spacers were trimmed using Cutadapt version 1.10 [94]. The mapping file was validated using Keemei [95]. QIIME2 version 2022.2 and the DADA2 plugin were used to de-noise and filter the reads, call amplicon sequence variants (ASVs), and generate a feature table [96, 97]. Taxonomy was assigned using the SILVA v138 99% database [98, 99]. Singleton sequences and sequences derived from chloroplasts or mitochondria were removed.

One vaginal microbiota sample was excluded from further analyses as it was mostly composed of Delftia, a known reagent contaminant. In the other samples, microbial DNA contamination did not have a significant effect, based on comparison of ASV prevalences and relative abundances in samples versus negative controls.

ASV data were analyzed using the R packages phyloseq 1.44.0 [100], mia 1.8.0 [101] and microbiome 1.23.1 [102]. Missing low-level taxonomic annotations (NA in phyloseq) were replaced with upper-level annotations using fantaxtic 2.0.1 [103]. Alpha diversity indices were calculated without rarefaction; rarefaction had no marked effect on the results. Statistical significances of alpha diversity differences were evaluated using the non-parametric Kruskal-Wallis rank-sum test and pairwise Wilcoxon rank-sum exact test with Bonferroni correction. Principal coordinates analyses, PERMANOVA and dbRDA were performed using vegan 2.6–6.14 [104], and pairwise PERMANOVAs using pairwiseAdonis version 0.4 [105]. Genus-level differential abundances for each sample type were calculated using MaAsLin 2 [106] (with 40% minimum prevalence and 0.5% minimum relative abundance, except 0.1% for rectal samples due to their high alpha diversity) and ANCOMBC [107] (without prevalence or abundance thresholds), with parity group and farm as fixed factors. The core microbiota heatmaps were generated using the microbiome package. Scater 1.18.6 package [108] was used to generate the violin plots. Venn diagram was generated using Venny 2.1 [109]. Color-blind friendly palettes were obtained from I Want Hue [110].

Species-level identifications were confirmed by a search of the corresponding ASV sequences against nucleotide sequence database using BLASTN 2.14.1+ [111]. Only identifications with 100% match with the specific species and no matches with > 98% similarity to any other species were considered valid.

## Supporting information

S1 Table Overview of the sequencing data. (PDF)

S2 Table Observed and expected compositions of the ZymoBiomics Microbial Community Standard. (PDF)

S3 Table Species-level comparison of the oral, vaginal, rectal and colostrum microbiota, Venn diagram of shared species. (XLSX)

S4 Table Parity group DbRDA and statistics summary. (XLSX)

S1 Fig Alpha diversity indices Shannon, Inverse Simpson, Faith, and observed richness of oral, vaginal, rectal and colostrum microbiota. (PDF)

S2 Fig Alpha and beta diversities of primiparous vs multiparous sows. (PDF)
