# Supplementary material for: Bacterial profiles of the oral, vaginal, and rectal mucosa and colostrum of periparturient sows
Source: PLoS One. 2025 Feb 12;20(2):e0317513. doi: 10.1371/journal.pone.0317513 (PMC11819496; doi:10.1371/journal.pone.0317513)
Supplement: S1 Table — (PDF) [file pone.0317513.s001.pdf]

Supplementary S1 Table

Piirainen et al. Bacterial profiles of the oral, vaginal, and rectal mucosa and colostrum of periparturient sows

S1 Table. Overview of the sequencing data.

Sample type	n	Nr of reads (Average ± SD)	Asv (Average ± SD)
Oral	31	49570 ± 9217	339 ± 71
Vaginal	31	61513 ± 9687	351 ± 221
Rectal	32	44881 ± 6380	625 ± 172
Colostrum	31	58722 ± 15543	404 ± 188
