# Supplementary material for: Bacterial profiles of the oral, vaginal, and rectal mucosa and colostrum of periparturient sows
Source: PLoS One. 2025 Feb 12;20(2):e0317513. doi: 10.1371/journal.pone.0317513 (PMC11819496; doi:10.1371/journal.pone.0317513)
Supplement: S2 Table — (PDF) [file pone.0317513.s002.pdf]

Supplementary S2 Table

Piirainen et al. Bacterial profiles of the oral, vaginal, and rectal mucosa and colostrum of periparturient sows

S2 Table. Observed and expected compositions of the ZymoBiomics Microbial Community Standard

Observed					***Expected***	
**Taxon**	**Relabund (repl. 1)**	**Relabund (repl. 2)**	**Average**		**Taxon**	**Relabund**
Bacillus	20,4 %	20,7 %	20,5 %		*Bacillus subtilis*	17,4 %
Enterococcus	8,4 %	9,0 %	8,7 %		*Enterococcus faecalis*	9,9 %
Escherichia-Shigella	13,9 %	13,6 %	13,7 %		*Eschericia coli*	10,1 %
Lactobacillus_fermentum	14,8 %	13,1 %	14,0 %		*Lactobacillus fermentum*	18,4 %
Listeria	12,5 %	12,8 %	12,6 %		*Listeria monocytogenes*	14,1 %
Pseudomonas	4,3 %	4,6 %	4,5 %		*Pseudomonas aeruginosa*	4,2 %
Salmonella	12,5 %	12,2 %	12,4 %		*Salmonella enterica*	10,4 %
Staphylococcus	13,2 %	13,8 %	13,5 %		*Staphylococcus aureus*	15,5 %
Delftia	0,02 %	0,02 %	0,02 %			
Enterobacteriaceae (family)	0,00 %	0,06 %	0,03 %			
