# Supplementary material for: Bacterial profiles of the oral, vaginal, and rectal mucosa and colostrum of periparturient sows
Source: PLoS One. 2025 Feb 12;20(2):e0317513. doi: 10.1371/journal.pone.0317513 (PMC11819496; doi:10.1371/journal.pone.0317513)
Supplement: S1 Fig — (PDF) [file pone.0317513.s005.pdf]

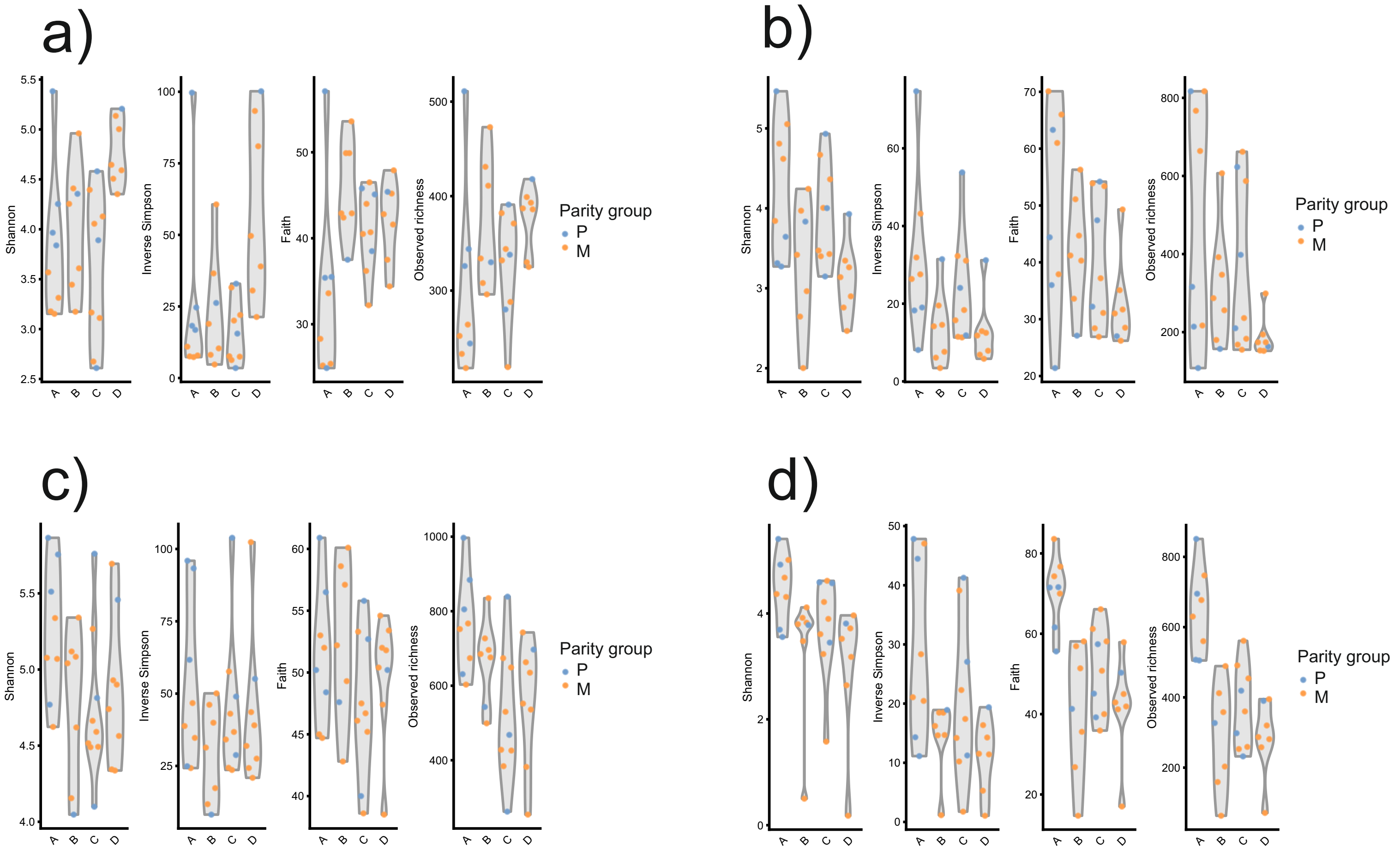

S1 Figure. Alpha diversity indices Shannon, Inverse Simpson, Faith, and observed richness of the a) oral, b) vaginal, c) rectal and d) colostrum microbiota of 32 late-pregnant sows from four Finnish commercial farms, marked as A-D.
