# Supplementary material for: Bacterial profiles of the oral, vaginal, and rectal mucosa and colostrum of periparturient sows
Source: PLoS One. 2025 Feb 12;20(2):e0317513. doi: 10.1371/journal.pone.0317513 (PMC11819496; doi:10.1371/journal.pone.0317513)
Supplement: S2 Fig — (PDF) [file pone.0317513.s006.pdf]

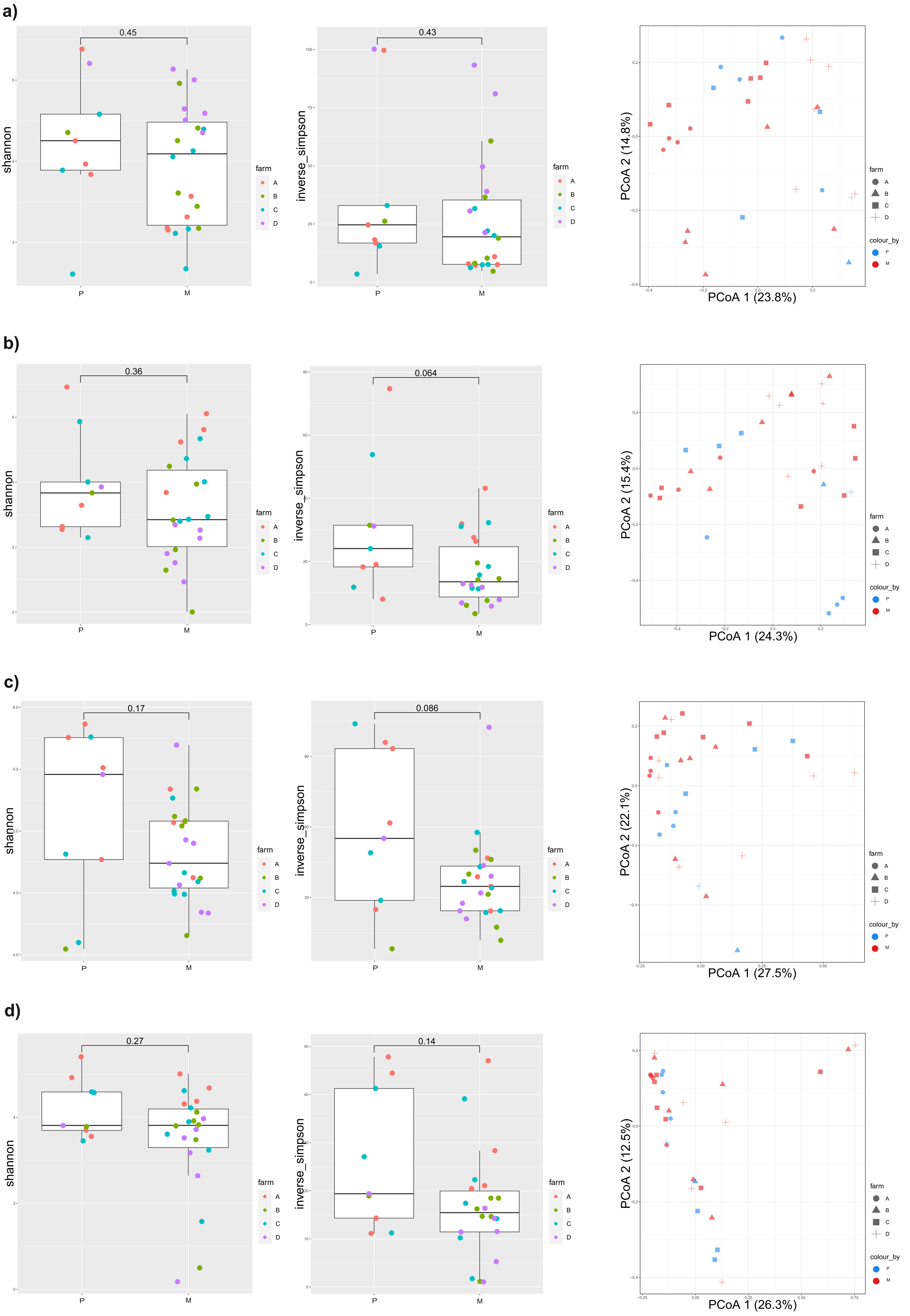

S1 Figure. Comparison or the alpha and beta diversities of a) oral, b) vaginal, c) rectal and d) colostrum microbiota of primiparous (P) and multiparous (M) sows. Alpha diversity index Shannon left, Inverse Simpson middle, differences between groups evaluated using non-parametric Kruskal-Wallis renk-sum test and pairwise Wilcoxon rank-sum exact test with Bonferroni correction, p values indcated by numbers above the boxplots. Principal components analysis based on Bray-Curtis dissimilarities left.
